# Processing of increased frequency of social interaction in social anxiety disorder and borderline personality disorder

**DOI:** 10.1038/s41598-021-85027-6

**Published:** 2021-03-09

**Authors:** Anna Weinbrecht, Michael Niedeggen, Stefan Roepke, Babette Renneberg

**Affiliations:** 1 Clinical Psychology and Psychotherapy, Freie Universität Berlin, Habelschwerdter Allee 45, 14195 Berlin, Germany; 2 Experimental Psychology and Neuropsychology, Freie Universität Berlin, Berlin, Germany; 3 Department of Psychiatry and Psychotherapy, Charité-Universitätsmedizin Berlin, Freie Universität Berlin, Humboldt-Universität zu Berlin, and Berlin Institute of Health, Campus Benjamin Franklin, Berlin, Germany

**Keywords:** Cognitive neuroscience, Emotion, Human behaviour, Psychiatric disorders

## Abstract

We investigated how patients with social anxiety disorder (SAD) and patients with borderline personality disorder (BPD) process an increase in the frequency of social interaction. We used an EEG-compatible version of the online ball-tossing game Cyberball to induce an increase in the frequency of social interaction. In the first condition, each player received the ball equally often (inclusion: 33% ball reception). In the following condition, the frequency of the ball reception was increased (overinclusion: 45% ball reception). The main outcome variable was the event-related potential P2, an indicator for social reward processing. Moreover, positive emotions were assessed. Twenty-eight patients with SAD, 29 patients with BPD and 28 healthy controls (HCs) participated. As expected, HCs and patients with BPD, but not patients with SAD, showed an increase in the P2 amplitude from the inclusion to the overinclusion condition. Contrary to our expectations, positive emotions did not change from the inclusion to the overinclusion condition. EEG results provide preliminary evidence that patients with BPD and HCs, but not patients with SAD, process an increase in the frequency of social interaction as rewarding.

## Introduction

To belong to a group is a central human need, which explains why interacting frequently with other individuals and feeling included into a group is important for our well-being^1,2^. In line with this, many studies have shown that being excluded from a group has detrimental effects on our well-being^3–5^. In this context, we were interested in the effects of changes in the quantity of social interaction: are there benefits when the frequency of social interaction is increased? This question is particularly interesting for individuals with social anxiety disorder (SAD), because individuals with SAD are afraid of embarrassing themselves in front of others and often try to avoid social interaction^6^. This study investigates how individuals with SAD process increased frequency of social interaction compared to individuals with borderline personality disorder (BPD) and healthy controls (HCs).

A possibility to investigate effects of increased frequency of social interaction provides the well-established virtual ball-tossing paradigm Cyberball^7^. During the Cyberball game, the participant believes that he/she is tossing the ball with two other co-players. However, the game is preprogrammed, so that it is possible to manipulate the frequency of ball reception. This allows to test the effect of inclusion (participant gets the ball as often as the co-player), exclusion (participant gets the ball less frequently), and overinclusion (participant gets the ball more frequently).

The effects of social exclusion on HCs have been examined in numerous Cyberball studies, for reviews see^8,9^, while the effects of social overinclusion have been less extensively examined^10–17^. In contrast to the negative effects of a transition to social exclusion^18–20^, a transition to overinclusion induces positive effects, like greater than anticipated enjoyment^17^ and a decrease in threat to fundamental social needs^13^. Notably, these effects were only reported when participants experienced a transition from inclusion to overinclusion^13,17^. The immediate onset of an overinclusion condition in the Cyberball game—not preceded by an inclusion condition—does not result in a beneficial effect for HCs^12,15^. Hence, exclusively the experience of an increase in the frequency of social interaction results in positive effects.

This study examines positive effects of the transition from social inclusion to social overinclusion. Self-report data can be biased by, for example, response tendencies, recall effects or social desirability^21^. To overcome these biases and to assess cognitive processes not covered by self-report data^22^, we recorded event-related brain potentials (ERPs) using an EEG-compatible version of the Cyberball game^18^.

To the best of our knowledge, only one Cyberball study examined the effects of the transition from social inclusion to social overinclusion relying on EEG data^13^. In this study, the transition from inclusion to overinclusion was associated with an increase in the frontal P2 amplitude^13^. The P2 amplitude is an ERP component, which has been related to the processing of rewarding stimuli^23–25^. For example, students showed a larger P2 amplitude when receiving positive compared to negative social feedback^26^. More precisely, the P2 amplitude has been related to the emotional evaluation of rewards^27,28^. This has been confirmed in recent studies across different experimental paradigms^29–31^. The P2 amplitude has also been related to other processes such as feature detection and allocation of attentional resources^32–34^ as well as emotional evaluation of stimuli^35,36^. However, we argue that in the context of the Cyberball paradigm the P2 amplitude is an indicator for reward processing, because none of these other processes can explain that the transition from inclusion to the overinclusion is associated with an increase in the P2 amplitude. For example, processes such as feature detection are not differently activated in the inclusion and the overinclusion condition. Moreover, attentional demands decrease in the overinclusion condition (the event of interest occurs more often) which should lead to a decrease in the P2 amplitude.

Importantly, next to the study by Niedeggen and colleagues^13^, another Cyberball study supported the notion that an increase in the frequency of social interaction is processed as socially rewarding with fMRI data: the transition from social inclusion to social overinclusion was associated with an activation of the ventral striatum, a region closely related to social reward processing^17^. Hence, previous Cyberball studies indicated that an increase in the frequency of social interaction serves as a social reward signal.

Which effect does an increase in the frequency of social interaction have on individuals with BPD and SAD? Research revealed that individuals with SAD are characterized by positivity impairments: they tend to process positive social information in a more negative way and tend to disqualify positive social information in a post-event process^37,38^. Hence, individuals with SAD might benefit less from an increase in the frequency of social interaction. In line with this, one Cyberball study with a non-clinical sample provided preliminary evidence that individuals high in social anxiety subjectively do not benefit from social overinclusion^39^. In this study, women high in social anxiety reported worse mood and less self-esteem in the overinclusion compared to the inclusion condition; a worsening of mood and self-esteem was not reported for women low in social anxiety. It could be speculated that the negative, external attributional style, which characterizes individuals with SAD^37,40,41^, contributes to these impairments in SAD.

Individuals with BPD are also highly impaired in social interactions^6^. They often act in an impulsive manner and easily feel excluded in social interactions^42,43^. Therefore, individuals with BPD might experience an increase in the frequency of social interaction as a protection from social exclusion and experience positive effects when socially overincluded. In line with this, a previous Cyberball study showed that participants with BPD experience reduced levels of negative mood in the overinclusion compared to the inclusion condition^44^. However, feelings of social belonging did not differ between conditions.

To summarize, previous Cyberball studies indicated that HCs experience positive effects from an increase in the frequency of social interaction. However, no study so far examined whether these positive effects also apply to individuals with SAD and individuals with BPD.

The current study seeks to close this gap and examines how participants with SAD process an increase in the frequency of social interaction compared to participants with BPD and HCs relying on EEG data. In a previous study, we focused on the analysis of expectancy processes in individuals with BPD and SAD. This process was tracked by the P3 component, which is related to context-updating processes^45,46^. In line with previous reports, individuals with BPD revealed a significant bias concerning the expected social involvement: independently of the actual participation (inclusion and overinclusion), the P3 signaled an expectancy violation. In line with the ERP data, participants with BPD felt more excluded^47^.

Whereas our previous analysis was focused on the expectancy-based processing of social participation in BPD, the current analysis focuses on the processing of social reward signals in SAD. As mentioned above, a corresponding ERP signature—a P2 component—can be elicited if a participant experiences the transition from social inclusion to overinclusion^13,25^.

We used a version of the Cyberball game established for EEG recording^18^. On a computer display, avatars of the participant and two co-players were displayed. Following the reception of the ball, the participant had the task to pass it to a co-player by pressing a corresponding button. In the first round of the Cyberball game, participants received the ball in 33% of the throws (inclusion). In the second round, the frequency of social interaction was increased and participants received the ball in 45% of all throws (overinclusion).

We hypothesized that the increase in the P2 amplitude from the inclusion to the overinclusion condition can be replicated in HCs and also applies to participants with BPD but does not apply to participants with SAD. Likewise, we hypothesized that HCs and participants with BPD, but not participants with SAD, report more positive emotions due to the transition from social inclusion to social overinclusion.

## Results

First, we confirmed that our experimental manipulation was successful: participants estimated to have received the ball more often in the overinclusion (*M* = 44.33%, *SD* = 17.31) than in the inclusion condition (*M* = 29.1%, *SD* = 10.84; *t*(81) = − 7.69, *p* < 0.001, *r* = 0.65).

### Change in P2 amplitude

**Figure 1:**
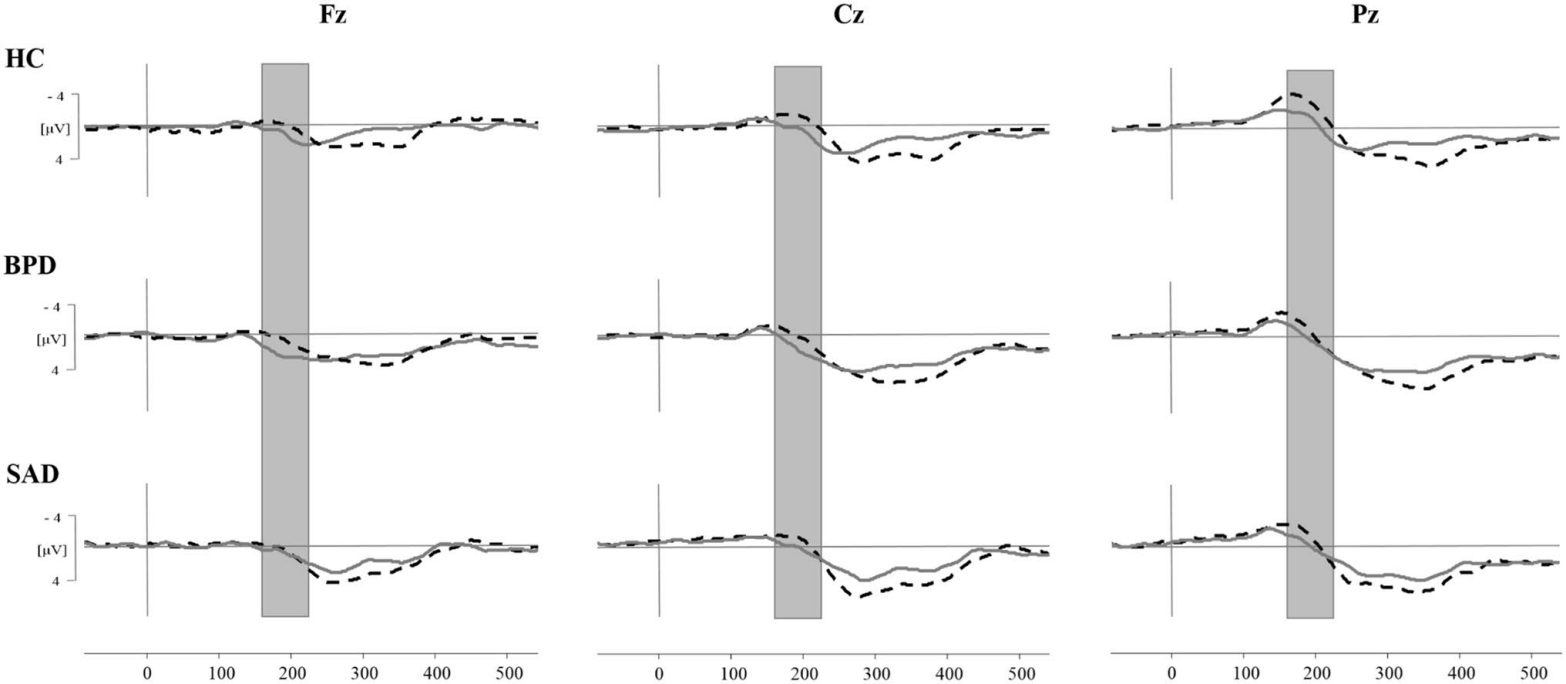
Grand averages of event-related potentials of each group at the frontal (Fz), central (Cz) and parietal (Pz) position. *Dashed black line *inclusion condition, *solid grey line *overinclusion condition, *HC *healthy controls, *BPD *borderline personality disorder, *SAD *social anxiety disorder. Amplitude differences between the conditions and groups were examined for the P2 time window at 160–225 ms (grey square).

Figure 1 depicts the grand-averaged ERPs for the three groups. The analysis focused on the time range from 160 to 225 ms: the P2 is defined as a frontally more positive-going wave in the overinclusion compared to the inclusion condition. This effect is markedly expressed in HCs and patients with BPD (see Fig. 1, left column). Means and standard deviations for the P2 amplitude are displayed in Table 1.

**Table 1:** Means and SDs of the P2 amplitude in social anxiety disorder, borderline personality disorder and healthy controls.

Condition	HC (*n* = 28)	SAD (*n* = 28)	BPD (*n* = 29)
	*M* (*SD*)	*M* (*SD*)	*M* (*SD*)
**P2 Fz**			
Inclusion	0.05 (3.56)	1.87 (3.18)	1.39 (4.19)
Overinclusion	2.04 (2.62)	1.76 (3.40)	3.40 (3.93)
**P2 Cz**			
Inclusion	− 0.33 (3.92)	2.03 (3.43)	2.09 (5.62)
Overinclusion	1.93 (2.87)	2.29 (3.53)	3.55 (4.93)
**P2 Pz**			
Inclusion	− 2.18 (3.52)	0.05 (4.11)	0.60 (5.23)
Overinclusion	0.40 (2.85)	0.95 (2.82)	1.47 (5.08)

*HC *healthy controls, *SAD* social anxiety disorder, *BPD *borderline personality disorder, *Fz *frontal, *Cz *central, *Pz *parietal.

The Greenhouse–Geisser corrected three-way interaction between “group”, “electrode position” and “condition” was significant, *F*(3.12) = 3.62, *p* = 0.01 (see supplementary information A for results of all lower order effects). We further explored this three-way interaction by focusing on the relevant interaction between “group” and “condition” separately for each electrode position. As expected, only at the frontal position (Fz), the change in the P2 amplitude between conditions differed between groups: the interaction between “group” and “condition” was significant at Fz (*F*(2) = 3.62, *p* = 0.03), but not at Cz (*F*(2) = 1.79, *p* = 0.17) and Pz (*F*(2) = 1.95, *p* = 0.15). Hence, we focused on the frontal position Fz for the Tukey corrected post-hoc analyses. In line with the visual inspection of Fig. 1 (left column), patients with BPD and HCs showed a significant increase in the P2 amplitude at the frontal electrode position from the inclusion to the overinclusion condition (HC: *t*(82) = − 3.11, *p* = 0.03, *r* = 0.32; BPD: *t*(82) = − 3.19,* p* = 0.024, *r* = 0.33), whereas patients with SAD did not (*t*(82) = 0.16, *p* = 1.00, *r* = 0.02). Results of group differences per condition can be found in supplementary information A.

#### Relation of the P2 component to later cognitive components

As mentioned in the introduction, a previous analysis focused on differences in the parietal P3 amplitude between groups to examine biases concerning the expected level of social involvement in BPD^47^. To test the assumption that the P3 amplitude—an indicator for expectancy violation—is related to a different information-processing step, we correlated the frontal P2 amplitude and the parietal P3 amplitude in all three groups. The result showed that the frontal P2 and parietal P3 amplitude were not significantly correlated in any of the groups (all Pearson's *r* < 0.25, all *p* > 0.19).

### Change in positive emotions

**Figure 2:**
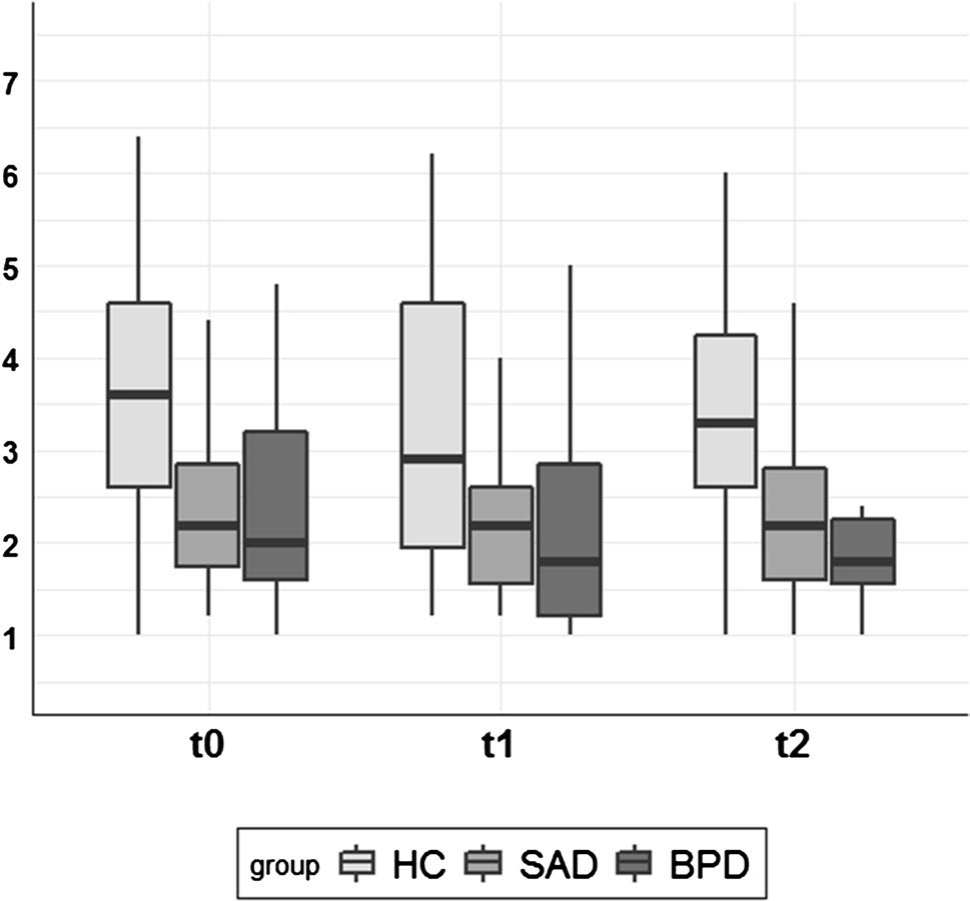
Box plots of positive emotions (range 0–7) for each group before the Cyberball game (t0), after the inclusion condition (t1) and after the overinclusion condition (t2). Boxes range from first to third quartile and represent the middle 50% of the data. Whiskers represent minimum and maximum scores. *HC *healthy controls, *BPD *borderline personality disorder, *SAD *social anxiety disorder.

Figure 2 displays positive emotions for each group before the Cyberball game (t0), after the inclusion condition (t1) and after the overinclusion condition (t2). Positive emotions changed over time (*F*(2) = 5.74, *p* = 0.004) and differed between groups (*F*(2) = 8.93, *p* < 0.001). However, positive emotions did not change differently over time in each group (*F*(4) = 0.52, *p* = 0.73). Note that the assumptions of homogeneity of variance and sphericity were violated. Therefore, we repeated the analyses within a multi-level model, which did not change results (see https://osf.io/sqgbr/?view_only=917a4a545f144ddd948c4ae6a3bcb2e5).

The Tukey corrected post-hoc analyses revealed that positive emotions decreased from before the Cyberball game to after the inclusion condition (t0 to t1: *t*(162) = − 3.38, *p* = 0.003, *r* = 0.26), but did not change significantly from after the inclusion to after the overinclusion condition (t1 to t2: *t*(162) = 1.94, *p* = 0.13, *r* = 0.15). Moreover, both clinical groups reported less positive emotions than HCs (HC vs SAD: *t*(81) = − 3.68, *p* = 0.001, *r* = 0.38; HC vs BPD: *t*(81) = − 3.64,* p* = 0.001, *r* = 0.37), while clinical groups did not differ from each other (*t*(81) = − 0.03, *p* = 1.00, *r* = 0.003).

Means and standard deviations for self-report data (positive emotions as well as self-focused and other-focused negative emotions) can be found in Table S1 in supplementary information B. Results of the ANOVA on self-focused and other-focused negative emotions can also be found in supplementary information B. Note that internal consistency was questionable for both negative emotions scales (see “Methods”) and results have to be interpreted with caution.

### Secondary analysis: differences in the attribution of increased frequency of the social interaction

Previous studies showed that individuals with SAD are characterized by a negative, external attributional style^37,40,41^. Therefore, in an exploratory analysis, we examined whether patients with SAD attributed the reason for receiving the ball more often in the second round differently than both other groups (see Table 2). Four possible attributions were provided: internal, chance or external, co-players’ dislike of each other, and co-players’ consideration. Groups differed in the extent to which they thought they received the ball more often in the second round because the other co-players didn’t like each other, but did not differ on the other three predetermined possible attributions (see Table 2). The post-hoc analyses revealed that patients with SAD attributed the reason for being overincluded more strongly to the co-players’ dislike for each other than HCs (*t*(79) = − 2.99, *p* = 0.01, *r* = 0.32) and patients with BPD (*t*(79) = 2.94,* p* = 0.01, *r* = 0.31) did. Patients with BPD and HCs did not differ in their attribution, *t*(79) = − 0.08,* p* = 1, *r* = 0.01.

**Table 2:** Results for attribution of the increased frequency of social interaction.

	HC	SAD	BPD	ANOVA	
	*M* (*SD*)	*M* (*SD*)	*M* (*SD*)	*df*	*F*
Internal	2.18 (1.09)	1.96 (1.17)	1.68 (1.25)	2, 81	1.28
Chance	2.57 (1.45)	3.26 (1.40)	2.61 (1.55)	2, 80	1.90
Co-players’ dislike	1.37 (0.69)	2.19 (1.30)	1.39 (0.92)	2, 79	5.84*
Co-players’ consideration	2.25 (1.04)	2.46 (1.23)	2.38 (1.52)	2, 82	0.20

*HC* healthy controls, *SAD *social anxiety disorder, *BPD *borderline personality disorder.

*p < 0.01.

## Discussion

This study examined how individuals with SAD, BPD and healthy individuals process an increase in the frequency of social interaction in a virtual ball tossing game (Cyberball) based on EEG data. As expected, healthy individuals and individuals with BPD, but not individuals with SAD, showed an increased P2 amplitude in transition from social inclusion to overinclusion. This provides preliminary evidence that individuals with SAD evaluate an increase in the frequency of social interaction as less rewarding than the other two groups. However, groups reported no changes in positive emotions due to the increased frequency of social interaction. In the following sections, results are discussed in more detail as well as embedded into the context of previous findings.

Our data confirmed the P2 effect in healthy individuals playing Cyberball: we replicated that the transition from social inclusion to overinclusion induces an increase in the P2 amplitude^13^. This indicates that healthy individuals may evaluate the increased frequency of social interaction as socially rewarding. As expected, this replicable P2 effect in healthy controls also applied to participants with BPD.

Our finding that individuals with SAD might not process increased frequency of social interaction as rewarding is in line with the impaired positivity hypothesis in SAD^37,38^. According to this hypothesis, individuals with SAD process and experience positive social information in a more negative way. In the context of social reward processing, Cremers and colleagues showed that individuals with SAD might lack a motivational drive to obtain a social reward, which was indicated by less striatal activity^48^. Moreover, Cao and colleagues reported that compared to healthy controls individuals with SAD show a smaller P2 amplitude when getting negative *or* positive social feedback^26^. These results are in line with the idea that social anxiety may impair the experience of social reward.

Both clinical groups reported less positive emotions than healthy participants did. This is in line with previous findings that individuals with SAD^49,50^ and BPD^51,52^ experience less positive emotions than healthy individuals do. However, changes in positive emotions did not reflect EEG results: positive emotions did not change from social inclusion to overinclusion. This contrasts the results of a previous Cyberball study, in which participants reported greater than anticipated enjoyment due to increased frequency of social interaction^17^. Moreover, this seems to contrast our interpretation that the P2 amplitude indicates reward processing. However, it has to be kept in mind that EEG data was assessed continuously throughout the Cyberball game, while positive emotions were assessed retrospectively after each condition. Hence, the different timing of assessment might have influenced our results. All in all, more studies are needed to investigate the effect of the transition from social inclusion to overinclusion on the P2 amplitude and on positive emotions.

However, our exploratory analyses provided preliminary evidence that the type of attribution might explain why specifically participants with SAD seem to benefit less from the transition to social overinclusion. Compared to participants with BPD and HCs, participants with SAD attributed the reason for the increased frequency of social interaction more strongly to an external factor: the co-players’ dislike for each other. It is known that individuals with SAD tend to interpret ambiguous social events as more negative and tend to disqualify positive social events in a post-event process^37^. Hence, the external attributional style in individuals with SAD might have disqualified the positive aspects of more social interaction^40^. Future research should examine the association between reward processing, social anxiety and attributional style.

Next, strengths and limitations of this study will be summarized. The strengths of this study are twofold. First, we examined differences in processing of social overinclusion in two clinical groups compared to a healthy control group. This highlights the specificity of altered cognitive processing in SAD. Second, EEG data provide a simultaneous measurement of the evaluation of social interaction and monitor processes not covered by self-report data^22^. Several limitations need to be mentioned: first, we only examined effects of the transition from social inclusion to overinclusion and did not randomize order of conditions. Second, we did not corroborate the EEG data with self-report data directly linked to the experience of social reward. Third, our exploratory analyses pointed towards the importance of an external attributional style in SAD. However, other underlying factors such as deviations in motivational preference for social reward^48^ might have also influenced the P2 effect. Fourth, we have to consider that the ERP effect might also be related to other processes, because the P2 effect is not selective for social reward processing (see “Introduction”). This is especially important, as the increase in the P2 amplitude was not associated with an increase in positive emotions in our study. However, as argued in the introduction, other cognitive processes that are associated with the P2 amplitude (e.g., feature detection, attentional processes) cannot explain the increase of the P2 amplitude from the inclusion to the overinclusion condition. Furthermore, we can rule out that the P2 amplitude is directly related to expectancy-related processes reflected in the P3 amplitude. Nevertheless, these limitations underline the importance of future research on the P2 effect in the context of the Cyberball paradigm.

To conclude, we replicated previous findings^13,17^ that healthy individuals show an increase in the P2 amplitude in the transition from social inclusion to overinclusion. This might indicate that healthy individuals process increased frequency of social interaction as rewarding. Importantly, we showed that this process can also be observed in individuals with BPD, but not in individuals with SAD. However, these results were not reflected in self-reported positive emotions. Future studies are needed to examine the P2 effect in the Cyberball paradigm.

## Methods

The current data were derived within a larger project on processing of social participation in BPD and SAD. Data on the bias in processing of social participation was published previously^47^.

The study was approved by the ethics committee of Freie Universität Berlin (ID 97 II /2016). The study was conducted in compliance with national legislation and the Code of Ethical Principles for Medical Research Involving Human Subjects of the World Medical Association (Declaration of Helsinki). All participants provided written informed consent.

### Participants

Overall, we included 85 participants in our analyses (identical to the sample in Weinbrecht et al.^47^): 28 HCs, 28 patients with SAD and 29 patients with BPD. All three groups were matched on age, IQ and gender (all *p* > 0.6). Participants were on average 28 years old (*SD* = 5.64) and mostly female (83.53%). Patients had on average 1.46 (*SD* = 1.18) comorbid diagnoses. The most common comorbid diagnosis was a remitted depressive disorder (total = 38.60%; SAD = 28.57%, BPD = 48.28%). Eight patients had a current mild depression (total = 14.04%; SAD = 21.43%, BPD = 6.90%). Fisher’s exact test revealed that patient groups did not differ in the number of comorbid current (*p* = 0.14) or remitted depressive disorders (*p* = 0.18). Antidepressant medication was taken by 29.83% of the patients (SAD = 25.00%, BPD = 34.48%).

Inclusion criteria for all participants were ages between 18 and 40 years. Exclusion criteria were mental retardation, epilepsy or organic brain disease, any psychotic disorder, current substance abuse/dependency, and intake of psychotropic medication within the last 4 weeks (antidepressant medication without any changes in the dose in the last 4 weeks was allowed). Note that we did not exclude participants with mutual comorbidity. One participant with BPD had a comorbid SAD diagnosis. Excluding this participant from the analyses did not change results.

Participants were recruited via media advertisement, the Department of Psychiatry of Charité Berlin and two university outpatient clinics in Berlin. Clinical psychologists, who were trained and supervised, confirmed DSM-IV diagnoses with the German versions of SCID I and SCID II^53^. Thirty patients (52.63%) were in ongoing psychiatric/psychotherapy treatment and had recently received a structured clinical interview. In these cases, DSM-IV diagnoses were available, and no additional diagnostic interview was conducted.

### Materials

#### Cyberball paradigm

Cyberball is a virtual ball-tossing game, in which the participant believes that he/she is tossing a ball with two other co-players^7^. The participant sits in front of a computer screen, on which he/she and the other two co-players are represented as avatars. Players can pass the ball to each other by pressing a corresponding button. However, the co-players are computer-generated, so that it is possible to manipulate how often the participant receives the ball from the co-players. We used the EEG-compatible version of Cyberball^18^ to manipulate the frequency of social interaction. In the first round, participants received the ball in 33% of the throws (inclusion condition). In the following round, participants received the ball in 45% of all throws (overinclusion condition). Each block consisted of 200 throws. The duration of the Cyberball task was about 14 min. Like most Cyberball studies, we used a cover story that informs the participant that Cyberball aims to test visual imagination capabilities. Participants rated the cover story to be plausible (*M* = 2.67, *SD* = 1.16).

The Cyberball game was presented on a computer screen (7° × 7° at a viewing distance of 140 cm) on which the avatars of the participant and the two putatively connected co-players were displayed. To indicate ball possession, the ball appeared in front of the avatar. When the participant decided to pass to one of the co-players, he/she had to press a corresponding button. Then, the ball appeared at a central position for 500 ms and next to the co-player for 500–2500 ms.

#### Questionnaires

##### Emotion Scale^54,55^

The Emotion Scale is a 14-item self-report inventory, which enables the assessment of positive emotions as well as self-focused negative and other-focused negative emotions. Participants rate on a 7-point scale (1 = not at all, 7 = very strongly) how much they experience a specific emotion at the moment. Mean scores are calculated for each scale: positive emotions (amusement, affection, contentment, pride), self-focused negative (loneliness, hurt, despair, sadness, fear, shame, guilt), and other-focused negative emotions (contempt, anger, resentment). Internal consistency was good for positive emotions (Cronbach’s α = 0.83–0.87). For self-focused negative (α = 0.64–0.85) and other-focused negative emotions (α = 0.49–0.69) internal consistency was questionable^56^.

##### Manipulation check

Participants had to estimate the percentage of ball tosses received per condition (open question) and the extent to which they believed in the cover story (range 1—5). The manipulation check questionnaire also included four items assessing the participants’ attribution of the increased frequency of social interaction in the second Cyberball round. Four possible attributions were provided (range 1–5): (1) internal (due to oneself), (2) chance, or external, (3) co-players’ dislike of each other, (4) co-players’ consideration.

#### EEG recording and data preparation

We recorded EEG data during the Cyberball game at three positions: frontal (Fz), central (Cz) and parietal (Pz) positions. Previous research provided evidence that these positions along the midline are sufficient to record the component of interest^13,45^. Moreover, focusing on these electrode positions allowed us to compare the pattern of results with previous studies using the same electrode montage^13,45^. Biosignals were recorded continuously with a sampling rate of 250 Hz.

We used Ag/AgCl electrodes, which were filled with electrode cream (Abralyt 2000, EASYCAP). Electrodes were embedded in an electrode cap (EASYCAP, Herrsching, Germany) to make sure positions were consistent across participants. Electrodes attached to the earlobes (impedance < 10 kΩ) served as the reference electrodes, with FCz serving as ground. Vertical and horizontal electrooculogram (EOG) were recorded to control for ocular artifacts (< 20 kΩ).

The onset of a ball possession (participant, co-player) was marked by a trigger signal. Offline, the EEG signal was segmented based on this trigger signal (− 200 to 600 ms epoch length) and then these EEG segments were baseline corrected (− 150 to 50 ms) and filtered (0.3–30 Hz band pass filter and 50 Hz notch filter). Artifacts (muscular or ocular artifacts, high alpha activity) were manually identified and excluded. The number of segments for the event “self overinclusion” was matched to the number of segments for the event “self inclusion” to ensure comparable signal-to-noise ratios. Participants in whom the averaged signal was based on less than 15 segments per condition following artifact rejection were excluded (in total 10 participants: 4 BPD, 1 SAD, 5 HC), leading to the sample of 85 participants as described above. The analysis focused on all events, in which the participant received the ball (self).

Averages for each participant were calculated, separately for condition (inclusion, overinclusion) and electrode position (frontal, central, parietal). Afterwards, grand averages were calculated for the P2 time window (average amplitude in the time frame from 160–225 ms), separately for the three groups (HC, SAD, BPD). The P2 time window for analysis was determined based on the grand averages of the ERPs. A corresponding time window was determined in the previous Cyberball study on the P2 effect^13^.

### Procedure

This study was part of a larger project^47,57^. Therefore, participants completed a web-based battery of questionnaires before the lab session. At the lab, we conducted clinical interviews if no diagnostic information was available. Electrodes were attached and participants completed a subcomponent of the “Leistungsprüfungssystem” (performance assessment system)^58^ to measure IQ. Participants played two blocks of Cyberball: first, all participants played the inclusion (33% ball possession) and afterwards the overinclusion condition (45% ball possession).

The study by Niedeggen und colleagues revealed that only *the transition from inclusion to overinclusion* is associated with the P2 effect^13^: when they played the inclusion condition first, healthy participants showed a larger P2 amplitude in the overinclusion condition, but not when they played the overinclusion condition first. Based on this previous result, we examined the transition from inclusion to overinclusion. Hence, we did not randomize order of conditions, which allowed us to obtain statistical power.

Each block consisted of 200 throws and lasted about 7 min. Participants answered the Emotion Scale before the Cyberball game (t0), after the inclusion condition (t1), and after the overinclusion condition (t2). After the Cyberball game (t2), participants also answered the manipulation check questionnaire. At the end of the lab session, participants were debriefed and signed informed consent again.

### Statistical analysis

We performed a mixed ANOVA on the P2 amplitude. Independent variables were the between-subject factor *group* (3 levels: HC, SAD, BPD) and the within-subject factors *condition* (2 levels: inclusion, overinclusion) and *electrode position* (3 levels: Fz, Cz, Pz). Furthermore, we performed a mixed ANOVA on positive emotions. Independent variables were the between-subject factor *group* (3 levels: HC, SAD, BPD) and the within-subject factors *time* (3 levels: t0, t1—after the inclusion condition, and t2—after the overinclusion condition). We further examined significant interaction effects with Tukey corrected post-hoc analyses. Pearson’s *r* was used as an effect size measure (small effect: *r* = 0.10; medium effect: *r* = 0.30; large effect: *r* = 0.50).

Analyses were conducted using R version 4.0.0^59^ and jamovi version 1.1.9.0^60^. An alpha level of 0.05 was applied.

## Supplementary Information

Supplementary Information.

## Data Availability

Data set and R syntax are available at https://osf.io/sqgbr/?view_only=917a4a545f144ddd948c4ae6a3bcb2e5.
