# Supplementary material for: Processing of increased frequency of social interaction in social anxiety disorder and borderline personality disorder
Source: Sci Rep. 2021 Mar 9;11:5489. doi: 10.1038/s41598-021-85027-6 (PMC7970905; doi:10.1038/s41598-021-85027-6)
Supplement: Supplementary file 1 — Supplementary Information. [file 41598_2021_85027_MOESM1_ESM.pdf]

**Supplementary Information**

# Processing of increased frequency of social interaction in social anxiety disorder and borderline personality disorder

**Authors:** Anna Weinbrecht[a], Michael Niedeggen[b], Stefan Roepke[c], Babette Renneberg[a]

**Affiliations:** [a]Clinical Psychology and Psychotherapy, Freie Universität Berlin, Berlin, Germany

[b]Experimental Psychology and Neuropsychology, Freie Universität Berlin, Berlin, Germany

[c]Department of Psychiatry and Psychotherapy, Charité – Universitätsmedizin Berlin, corporate member of Freie Universität Berlin, Humboldt-Universität zu Berlin, and Berlin Institute of Health, Campus Benjamin Franklin

**Supplementary Information A: Detailed results of the ANOVA on the P2 amplitude**

Significant main effect of “condition” $F(1) = 13.01$, $p < 0.001$) and of the Greenhouse-Geisser corrected main effect of “electrode position” ($F(1.42) = 27.99$, $p < 0.001$). Main effect of “group” was not significant, $F(2) = 2.23$, $p = 0.11$. The Greenhouse-Geisser corrected two-way interactions between “group” and “electrode position” ($F(2.84) = 0.45$, $p = 0.71$) as well as between “condition” and “electrode position” ($F(1.56) = 0.22$, $p = 0.75$) were not significant. The two-way interactions between “group” and “condition” ($F(2) = 2.18$, $p = 0.12$) was also not significant. As described in the main text, the Greenhouse-Geisser corrected three-way interaction between “group”, “electrode position” and “condition” was significant, $F(3.12) = 3.62$, $p = 0.01$. Most importantly, the relevant interaction between “group” and “condition” was only significant at Fz ($F(2) = 3.62$, $p = 0.03$), but not at Cz ($F(2) = 1.79$, $p = 0.17$) and Pz ($F(2) = 1.95$, $p = 0.15$).

Main effects at the frontal position: Main effect of “group” ($F(2) = 2.40$, $p = 0.10$) and “condition” ($F(1) = 0.47$, $p = 0.50$) were not significant.

Results of Tukey corrected post-hoc analyses of differences between groups per condition at the frontal position: no significant differences between groups in the inclusion (all $p > 0.39$) or in the overinclusion condition (all $p > 0.50$).

Results of Tukey corrected post-hoc analyses of differences between conditions per group at the frontal position: No significant differences for patients with SAD ($t(82) = 0.16$, $p = 0.87$, $r = 0.02$). Significant differences for patients with BPD ($t(82) = -3.19$, $p = 0.002$, $r = 0.33$) and HCs ($t(82) = -3.11$, $p = 0.003$, $r = 0.32$).

## Supplementary Information B: Emotion Scale

*Table S1: Means and SDs of emotion subscales (positive emotions, self-focused negative and other-focused negative emotions) in social anxiety disorder, borderline personality disorder and healthy controls.*

| | | HC ($n$ = 28) | SAD ($n$ = 28) | BPD ($n$ = 29 [a]) |
|---|---|---|---|---|
| | Time | *M* (*SD*) | *M* (*SD*) | *M* (*SD*) |
| Positive emotions | t0 | 3.68 (1.36) | 2.42 (0.98) | 2.45 (1.13) |
| | t1 | 3.21 (1.46) | 2.18 (0.78) | 2.26 (1.21) |
| | t2 | 3.39 (1.33) | 2.38 (1.03) | 2.37 (1.52) |
| Negative self-focused emotions | t0 | 1.15 (0.31) | 1.73 (0.74) | 2.40 (1.30) |
| | t1 | 1.14 (0.24) | 1.44 (0.67) | 2.10 (1.46) |
| | t2 | 1.08 (0.18) | 1.29 (0.44) | 1.97 (1.27) |
| Negative other-focused emotions | t0 | 1.23 (0.37) | 1.44 (0.50) | 1.94 (1.14) |
| | t1 | 1.33 (0.43) | 1.56 (0.52) | 2.14 (1.16) |
| | t2 | 1.31 (0.47) | 1.42 (0.54) | 1.86 (0.85) |

*Note*. HC = healthy controls, SAD = social anxiety disorder, BPD = borderline personality disorder. t0 = before the Cyberball game, t1 = after the inclusion condition, t2 = after the overinclusion condition

[a] at t2: n = 28

**Results of the ANOVA on self-focused and other-focused negative emotions**

Self-focused negative emotions:

Significant main effect of "time" ($F(2) = 8.59$, $p < 0.001$) and "group" ($F(2) = 11.80$, $p < 0.001$). The two-way interaction between "time" and "group" was not significant, $F(4) = 1.59$, $p = 0.18$.

Results of Tukey corrected post-hoc analyses of differences in self-focused negative emotions between groups: Patients with BPD experienced significant more self-focused negative emotions than patients with SAD ($t(81) = 2.98$, $p = 0.01$) and HCs ($t(81) = 4.82$, $p < 0.001$). Patients with SAD and HCs did not differ from each other, $t(81) = 1.83$, $p = 0.17$.

Results of Tukey corrected post-hoc analyses of differences in self-focused negative emotions over time: Negative self-focused emotions decreased from t0 to t1 ($t(162) = -2.95$, $p = 0.01$), but did not change from t1 to t2 ($t(162) = -1.05$, $p = 0.55$).

Other-focused negative emotions:

Significant main effect of "group" ($F(2) = 8.43$, $p < 0.001$). The main effect of "time" ($F(2) = 2.48$, $p = 0.09$) and the two-way interaction between "time" and "group" were not significant ($F(4) = 0.37$, $p = 0.83$).

Results of Tukey corrected post-hoc analyses of differences in other-focused negative emotions between groups: Patients with BPD experienced significant more other-focused negative emotions than patients with SAD ($t(81) = 2.86$, $p = 0.02$) and HCs ($t(81) = 3.98$, $p < 0.001$). Patients with SAD and HCs did not differ from each other, $t(81) = 1.13$, $p = 0.50$.
